# Solid-State Limited Nucleation of NiSi/SiC Core-Shell Nanowires by Hot-Wire Chemical Vapor Deposition

**DOI:** 10.3390/ma12040674

**Published:** 2019-02-24

**Authors:** Mahdi Alizadeh, Najwa binti Hamzan, Poh Choon Ooi, Muhammad Firdaus bin Omar, Chang Fu Dee, Boon Tong Goh

**Affiliations:** 1 Low Dimensional Materials Research Centre (LDMRC), Department of Physics, Faculty of Science, University of Malaya, Kuala Lumpur 50603, Malaysia; alizadeh_kozerash@yahoo.com (M.A.); najwahamzan@yahoo.com (N.b.H.); 2 Laser-Plasma Research Institute, Shahid Beheshti University, G.C., Evin, Tehran 19839, Iran; 3 Institute of Microengineering and Nanoelectronics, Universiti Kebangsaan Malaysia, Bangi 43600, Malaysia; pcooi@gmx.com (P.C.O.); deechangfu@gmail.com (C.F.D.); 4 Ibnu Sina Institute for Fundamental Science Studies (IIS), Universiti Technologi Malaysia, Skudai 81310, Johor, Malaysia; firdausomar@utm.my

**Keywords:** core-shell nanowires, NiSi, SiC, nucleation limited silicide reaction, surface-migration, hot-wire chemical vapor deposition (HWCVD)

## Abstract

This work demonstrated a growth of well-aligned NiSi/SiC core-shell nanowires by a one-step process of hot-wire chemical vapor deposition on Ni-coated crystal silicon substrates at different thicknesses. The NiSi nanoparticles (60 to 207 nm) acted as nano-templates to initially inducing the growth of these core-shell nanowires. These core-shell nanowires were structured by single crystalline NiSi and amorphous SiC as the cores and shells of the nanowires, respectively. It is proposed that the precipitation of the NiSi/SiC are followed according to the nucleation limited silicide reaction and the surface-migration respectively for these core-shell nanowires. The electrical performance of the grown NiSi/SiC core-shell nanowires was characterized by the conducting AFM and it is found that the measured conductivities of the nanowires were higher than the reported works that might be enhanced by SiC shell layer on NiSi nanowires. The high conductivity of NiSi/SiC core-shell nanowires could potentially improve the electrical performance of the nanowires-based devices for harsh environment applications such as field effect transistors, field emitters, space sensors, and electrochemical devices.

## 1. Introduction

One-dimensional (1D) semiconductor nanostructures such as nanorods, nanotubes and nanowires have recently showed exciting scientific challenges and technological applications especially in nanoscale sensors, optoelectronic devices, energy generators, and storage devices [1,2,3]. Highly metallic 1D NiSi nanowires possess excellent electrical, field emission and magnetic properties which make them a potential candidate for interconnectors, field emitters, functional micro-tips, biosensors, and micro-supercapacitors [4,5,6,7]. The superior ferromagnetic property with high coercivity of NiSi nanowires was reported to be attributed to its nanoscale and high Ni/Si ratio [8]. Moreover, their tiny nanoscale geometric feature and high aspect ratio, allow the nanowires to enhance the light-active region and provide a large surface area at a fixed volume for photovoltaic architecture [9,10]. The unique physical properties of cone-shape well-aligned NiSi nanowires has demonstrated an excellent field emission property with their low turn-on field as recently reported by Lin et. al. [11]. However, it has been reported that the silicide nanowires suffer from oxidation on their surfaces, which generally degrades their electrical properties such as charge collection and results in charge recombination [12,13]. Fabrication of core-shell nanowires is one of the most effective solution to overcome this intrinsic limitation of the intrinsic single nanowires [14]. SiC possesses high chemical stability, significant mechanical strength, high electron mobility and adjustable electrical conductivity [15,16,17], having capability to fulfill the requirements as a shell material. Therefore, the incorporation of SiC into the core-shell nanowires is motivated by its superior properties and furthermore, the NiSi/SiC core-shell nanowires could potentially open a new direction of research into energy generation and storage applications [7]. 

Hot-wire chemical vapor deposition (HWCVD) provides a one-step process for growth of NiSi/SiC core-shell nanowires by decomposing the source gases (SiH_4_ and CH_4_) simultaneously using hot-filament at a temperature above 1800 °C [18,19]. The formation of hybrid Si core-shell in 1D structure is unprecedented and rarely found in the literature to the best of our knowledge. Therefore, the Ni induced growth of NiSi/SiC core-shell structure could possess a synergetic effect, which may be inhibiting their intrinsic limitations [20]. Normally, the 1D nanowires growth is followed by the conventional vapor–liquid–solid (VLS) mechanism [21]. The nanowire growth in VLS process is driven by a metal catalyst droplet in liquid state at a temperature above the eutectic point of the two elements. However, the formation of NiSi nanowires works differently as compared to the VLS mechanism, which mainly involving the reaction of Ni and Si through solid state diffusion [22]. The growth of NiSi nanowires depends on the reaction of Ni and Si in a solid condition at a temperature much lower than the eutectic point of Ni and Si (approximately 962 °C) [23]. However, the dependence of core-shell nanowires growth on the NiSi particle size and Ni induction have not been studied in detail.

In this work, the NiSi/SiC core-shell nanowires were grown by a one-step process of HWCVD at different Ni thicknesses. The effects of the Ni thickness on the morphology, growth rate, solid catalyst particle size, and nanowire’s growth mechanism are well investigated. Moreover, the nanoscale electrical properties of the nanowires are demonstrated, which may have potential for nano-electronic and nano-optoelectronic applications. 

## 2. Experimental Methods

NiSi/SiC core-shell nanowires were grown on Ni-coated p-type crystal silicon (c-Si) with (100) plane substrates by a home-built HWCVD system [24]. The c-Si substrates were conventionally coated with 100 nm-thick SiO_2_ layer. The layer is used to prevent any diffusion of Ni into the substrates. The Ni films were deposited on the substrates using a coiled tungsten filament (99.95% purity) as a heating element. In order to form metal nanoparticles, the Ni films were subsequently treated by energetic atomic hydrogen in plasma ambient for 10 min. During the plasma treatment, the substrate temperature, deposition pressure, hydrogen gas flow-rate and radio-frequency power were fixed at 450 °C, 0.75 mbar, 100 sccm and 5 W, respectively. For the deposition purpose, the filament temperature and deposition pressure were maintained at 1850 °C and 3 mbar, respectively. The flow of SiH_4_, CH_4_, and H_2_ gases into the reaction chamber were controlled by mass-flow controllers at the flow-rates of 1, 2, and 100 sccm, respectively. The filament temperature was measured using a pyrometer model Reytek, Raynger 3i. The filament-to-substrate distance was kept at 2 cm with the deposition time was fixed at 5 min. The growth process was studied by varying Ni thickness (110, 190, and 270 ± 30 nm).

Field emission scanning electron microscopy (FESEM) secondary electron (SE) and backscattered electron (BSE) images of the nanowires were obtained using a Hitachi SU 8000 SEM (Tokyo, Japan) at electron accelerating voltages of 2 and 10 kV respectively. The elemental analysis on the samples were conducted by energy-dispersive X-ray (EDX) detector (Bruker XFlash6∣100, Tokyo, Japan) attached to the SEM, at an electron accelerating voltage of 15 kV. The working distances for imaging and EDX were fixed at 8 and 15 mm, respectively. The chemical compositions and bonding configuration of the nanowires were investigated by X-ray photoelectron spectroscopy (XPS, PHI Quantera II, ULVAC-PHI, Inc., Kanagawa, Japan). The XRD pattern was recorded over the 2θ range of 20 to 80° at a fixed grazing incidence angle of 0.5° using a PANalytical Empyrean X-ray diffractometer (Malvern Panalytical Ltd., Royston, UK) with X-ray wavelength of 1.5406 Å. The step time and step size of the scanning were fixed at 3.52 s and 0.026°, respectively. The cross-section BSE images of the nanowires were collected using a Bruker photodiode-backscattered electron (PDBSE) detector (Bruker, Tokyo, Japan). The chemical states of the samples were examined by scanning photoelectron microscopy (SPEM, NSRRC, Hsinchu, Taiwan) at the 09A1 beamline of the National Synchrotron Radiation Research Center (NSRRC), Hsinchu, Taiwan. Themonochromatic soft X-ray (*h* = 380 eV) was focused by a combination of Fresnel zone plate and order sorting aperture. The nanowires coated on the c-Si substrate was in-situ cleaved under UHV conditions in the SPEM chamber to keep a clean cross-section surfaces for the chemical imaging and micro-area photoelectron spectroscopy (PES, NSRRC, Hsinchu, Taiwan). The sample position relative to the focused photon beam was controlled by a piezo driven flexure stage (range: 10 × 10 µm^2^) for a raster scanning in SPEM imaging and fine positioning in the micro-area PES. The obtained binding energies (BE) were calibrated using the C 1s energy of 284.6 eV attributed to adventitious carbon that exhibits in all air-exposed materials. The TEM and high-resolution TEM images of the nanowires were collected using a TEM (JOEL JEM-2100F, JEOL Ltd., Tokyo, Japan) with an accelerating voltage of 200 kV. The dispersing nanowires for TEM measurement were prepared on carbon-coated copper grids (Lacey 300 mesh Cu). The EDX elemental mappings of a single nanowire were performed using STEM/High-angle annular dark-field (HAADF, JEOL Ltd., Tokyo, Japan) and Oxford EDX detectors (Oxford Instruments NanoAnalysis, High Wycombe, UK). The surface roughness and grain sizes of samples were characterized using Atomic Force Microscopy (AFM, SII NanoTechnology Inc., SI-DF-3R(100), Tokyo, Japan), with a silicon nitride cantilever (SI-DF3, Applied NanoStructures, Inc., Mountain View, CA, USA). The scanning was done by contact mode in a range of 10 μm × 10 μm. The current–voltage (I–V) curves of the nanowires were obtained by the AFM in conducting mode. 

## 3. Results and Discussion

**Figure 1:**
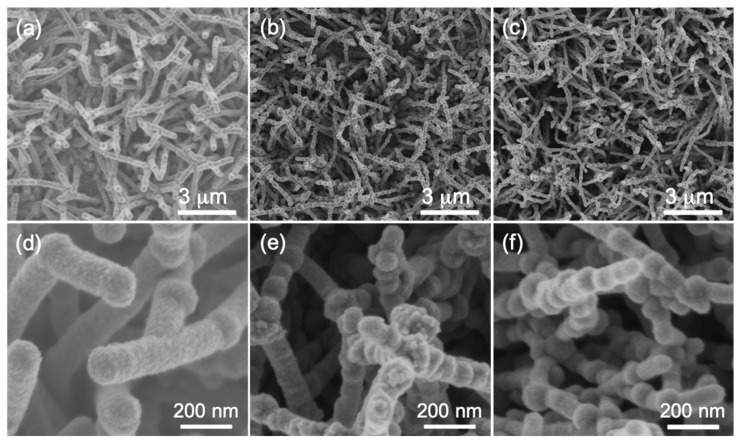
Field emission scanning electron microscopy (FESEM) images of the NiSi/SiC core-shell nanowires grown by hot-wire chemical vapor deposition (HWCVD) at different Ni thicknesses of (**a**,**d**) 110, (**b**,**e**) 190, and (**c**,**f**) 270 nm. The top and bottom images represent low and high magnifications of the micrograph images, respectively.

Figure 1 shows the FESEM images of the NiSi/SiC core-shell nanowires grown on c-Si substrates at different Ni thicknesses. These surface images were scanned in SE mode at low electron accelerating voltage of 2 kV. The nanowires are in high density and uniformly distributed on the substrate surfaces and it could be observed that the density of nanowires is directly proportional with the thickness of Ni as shown in Figure 1a–c. The surface morphology of the nanowires changes significantly with increase in Ni thickness as shown in Figure 1d–f. This can be clearly shown on the formation of bumps along their lengths and the number of bump increases with increase in Ni thickness. The formation of these bumps is generally deduced by the out-diffusion of NiSi core [25]. These bumps were reported to potentially grow nanowire branches for electrochemical electrode and sensing applications [26]. The nanowires grown at Ni thicknesses of 190 and 270 nm exhibit irregular and branched morphologies as shown in Figure 1d,e, respectively. The high surface diffusion of Ni in Si increases the degree of supersaturation for the nanowire growth and, thus, enhances the growth rate of the nanowires by increasing the axial growth of the nanowires [27]. The noticeable growth of branch nanowires at the high Ni thicknesses is obviously attributed to the out-diffusion of the NiSi core which leading to the secondary growth of the core-shell nanowires at the bump areas. The out-diffusion of the metal catalysts in the growth of the nanowires was also observed by Hannon et al. and occurred during the mediation of the axial growth of the core nanowire [28]. The lateral expansion of the NiSi core is happened to release the stress applied by the axial growth of the shell nanowire [25]. This leads to the out-diffusion and thus results in the secondary growth of the nanowire branches at the out-diffusion points. The estimated diameters of the trunk nanowires are approximately 144, 89, and 76 ± 15 nm for the nanowires grown at the Ni film thicknesses of 110, 190, and 270 nm, respectively. The morphology result clearly indicated that the thicker Ni film produces higher density and smaller diameter of NiSi/SiC core-shell nanowires grown by HWCVD at substrate temperature of 450 °C.

**Figure 2:**
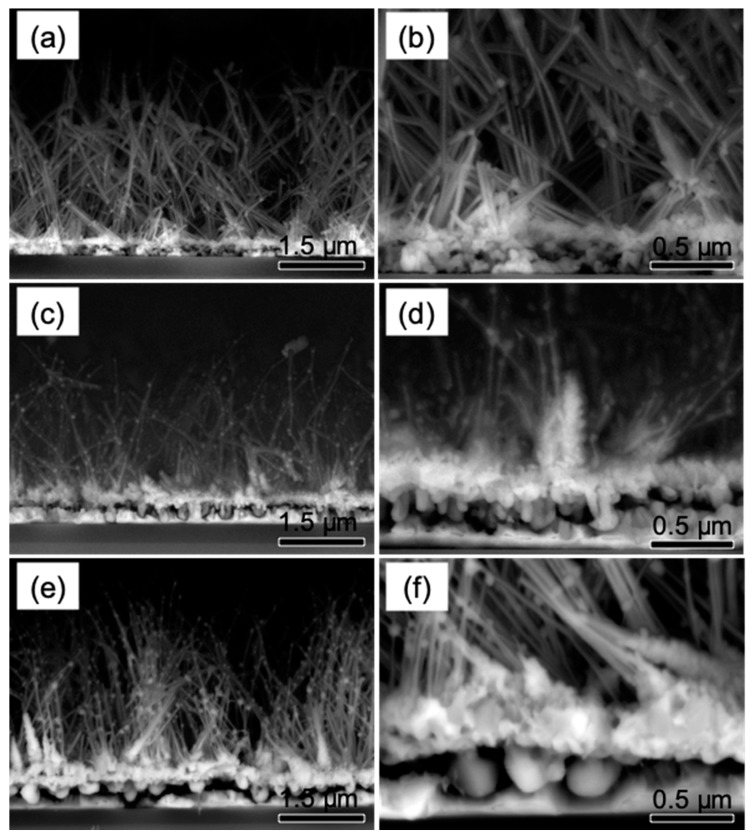
FESEM cross-section images of NiSi/SiC core-shell nanowires grown by HWCVD on c-Si substrate at different Ni thicknesses of 110, 190, and 270 nm. The FESEM images scanned at low magnification (**a**,**c**,**e**) and the respective images scanned at high-magnification (**b**,**d**,**f**) by using photodiode-backscattered electron (PDBSE) detector.

Figure 2 shows the FESEM cross-section images of the core-shell nanowires grown by HWCVD at different Ni film thicknesses. These images were obtained by collecting the BSE signals at electron accelerating voltage of 10 kV. The low magnification images in Figure 2a,c,e show that the nanowires are mostly straight with randomly aligned morphologies. The increase in the Ni thickness is likely improves the alignment of the nanowires towards vertically morphologies. However, the thicker Ni films reduce the height of the nanowires indicating that the Ni thickness significantly affect the growth rate of the nanowires. The estimated heights of the nanowires were approximately 3769 ± 549, 2532 ± 276, and 3351 ± 234 nm for the Ni film thicknesses of 110, 190, and 270 nm, respectively. Furthermore, some noticeable NiSi nanoparticles were clearly visible on the stems of the nanowires. The formation of these NiSi nanoparticles is probably attributed to the out-diffusion of NiSi core nanowires and the out-diffusion phenomena can be evidently depicted by the BSE images as shown in Figure 2b,d. In addition, the deposited layers under the nanowires were clearly observed for all the samples at different Ni thicknesses. These deposited layers were formed by the NiSi alloy solid particles as illustrated in the high magnification images (Figure 2b,d,f)). The spontaneous diffusion of Ni into the deposited Si layers forms the high density of NiSi solid particles [20]. Apparently, the increase in the thickness of the Ni film leads to the formation of larger NiSi solid particles. The estimated grain size from the images for the nanowires grown at the Ni film thicknesses of 110, 190, and 270 nm were about 60 ± 13, 107 ± 29, and 207 ± 35 nm respectively. The highest Ni film thickness shows an appearance of faceted crystals on the deposited layer as clearly depicted in Figure 2f.

**Figure 3:**
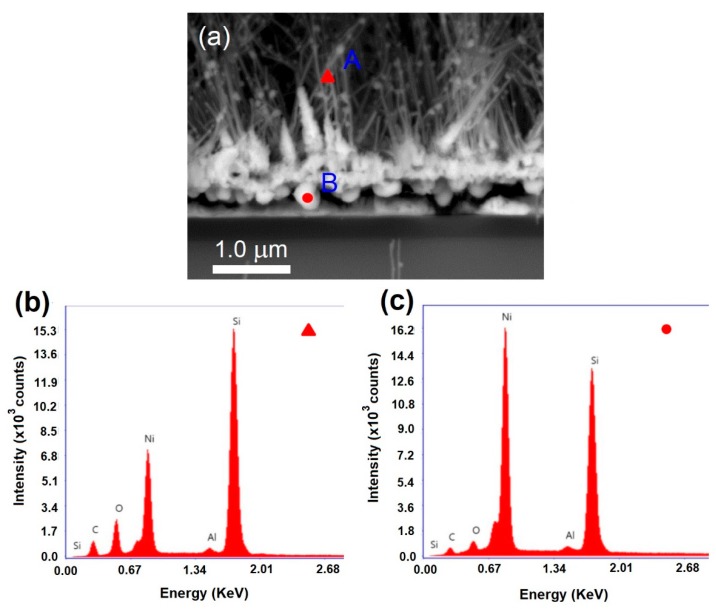
(**a**) The FESEM cross-section image of the nanowires scanned by PDBSE detector. (**b**,**c**) energy-dispersive X-ray (EDX) spectra obtained at the stem of nanowires (marked as A) and at the solid particle between the deposition layer and c-Si substrate (marked as B) respectively as labelled in (**a**).

Figure 3a depicts the FESEM cross-section image for the elemental analysis of EDX point scan on the stem of nanowires (A) and the NiSi solid particles under the deposited layer (B). The EDX spectra for the scans at A and B are shown in Figure 3b,c, respectively. Obviously, the point A shows relatively higher contents of C, O, and Si than point B except the Ni content which is in contrast and higher in point B. The elemental contents of the points A and B are tabulated in Table 1. Quantitatively, the stem of the nanowires consists of C (25.45%), O (13.72%), Ni (11.70%), and Si (48.03%) indicating typical elemental content of NiSi/SiC core-shell nanowire compositions. The presence of O content is likely inferred to the formation of thin amorphous SiO*_x_* on the nanowires due to the oxidation when the nanowires were exposed to the atmospheric ambient [29]. It is worth noting that the NiSi solid particle compositions were dominated by Ni (29.19%) and Si (45.14%) comparatively with C (17.02%) and O (7.46%). The presence of C and O are likely because of the deposition of SiC layer and oxide residuals or oxidation, respectively. The EDX results reveal that the NiSi alloy particles act as solid seeds for the spontaneous growth of NiSi core nanowires followed by the nucleation limited silicide reaction as modelled by Kim et. al. [30]. The successive spontaneous diffusion of Ni into the NiSi particles leads to the growth of NiSi nanowires once it reached to the degree of supersaturation. Further, the out-diffusion of Ni into the deposited Si layer increased the size of the NiSi particles. However, the radial deposition of SiC on the particles limited the further growth of the NiSi particles [31]. This significantly increases the volume free energy and consequently reduces the surface free energy leading to the growth of axial NiSi core nanowires. The radial deposition of SiC on the NiSi particles mediates as SiC shells is followed by the axial growth of NiSi core nanowires.

**Figure 4:**
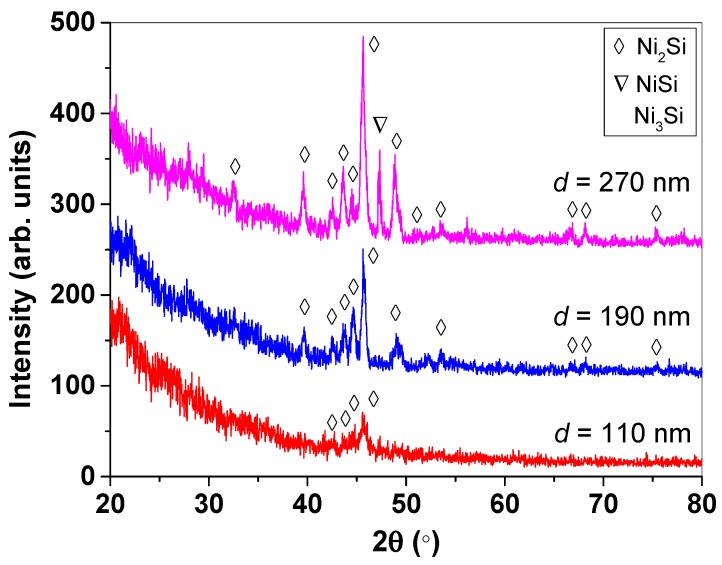
XRD patterns of the NiSi/SiC core-shell nanowires grown by HWCVD at different Ni thicknesses of 110, 190, and 270 nm. The legend indicates the phases of the NiSi chemical formula.

Figure 4 depicts the XRD pattern of the core–shell nanowires grown at different Ni thicknesses. At Ni thickness of 110 nm, only small crystalline peaks are appeared at 2θ = 42.729°, 43.535°, 44.575°, and 45.589°, which are corresponded to crystallographic planes of (310), (021), (220), and (121) orientations for Ni_2_Si phase according to the JCPDS card number of 048-1339. The relatively low intensities of the of Ni_2_Si peaks in the XRD pattern for the nanowires grown at Ni thickness of 110 nm is likely due to insufficient diffusion of Ni into the Si during the deposition. The nanowires grown at Ni thickness of 190 nm demonstrates two main phases of crystalline NiSi such as Ni_2_Si and Ni_3_Si phases. A single crystalline peak located at 2θ = 52.193° belongs to crystallographic plane of (200) orientation for Ni_3_Si phase based on JCPDS number of 065-3243. Whereas, the various crystalline peaks located at 2θ = 32.563°, 39.661°, 42.495°, 43.613°, 44.627°, 45.641°, 48.995°, 51.465°, 53.571°, 66.675°, 68.209°, and 75.463° are associated to crystallographic planes of (111), (211), (310), (021), (220), (121), (002), (221), (320), (312), (222), and (322) orientations for Ni_2_Si phase respectively, followed the JCPDS number of 048-1339. As the Ni thickness increases to 270 nm, the crystalline of Ni_2_Si phase is dominant accompanied with crystalline NiSi phase. A single crystalline peak of NiSi phase appeared at 2θ = 47.331° corresponds to crystallographic plane of (121) orientation according to the JCPDS number of 01-085-0901. The increase in Ni thickness leads to the formation of homogeneous crystalline Ni-rich Ni_2_Si core nanowires attributed to effectively diffusion of Ni into the NiSi particles which act as a nucleation site for the growth of these core-shell nanowires.

**Figure 5:**
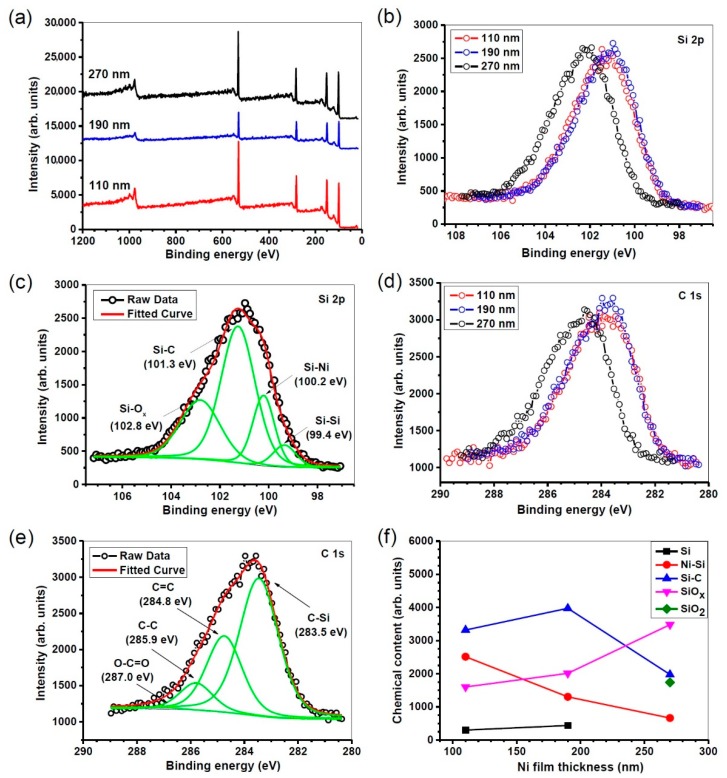
(**a**) XPS survey-scan spectra of the NiSi/SiC core-shell nanowires grown by HWCVD at different Ni thicknesses. (**b**,**d**) XPS high-resolution spectra of the nanowires grown at different Ni thicknesses for Si 2p and C 1s respectively. (**c**,**e**) Typical respective deconvoluted components for Si 2p and Ni 2p respectively. (**f**) Variations of chemical contents against Ni thicknesses which the contents were directly obtained from the integrated intensities of the deconvoluted components at Si 2p band.

The chemical compositions and bonding configuration of the samples were analyzed by XPS and the results are shown in Figure 5. Figure 5a depicts the XPS survey scan spectra of the NiSi/SiC core-shell nanowires grown at different Ni thicknesses. The Si 2s, Si 2p, C 1s, and O 1s peaks are clearly presented in the spectra. The Si 2p and C 1s core-level photoelectron spectra of the nanowires grown at different Ni thicknesses are shown in Figure 5b,d, respectively. It can be seen that for the nanowires grown at Ni thickness of 270 nm, the Si 2p and C 1s core-level photoelectron spectra are shifted toward higher photon energies. This reveals a variation in the compositions of the nanowires grown at highest Ni thickness. To further elaborate this variation, the Si 2p and C 1s band spectra were deconvoluted into several Si and C-related components as depicted in Figure 5c,e, respectively. The deconvolution of the Si 2p peak shows that its consists of four major components at 102.8, 101.3, 100.2, and 99.4 eV, which are attributed Si–O_x_, Si–C, Si–Ni, and Si–Si bonds, respectively. The C 1s peak also was deconvoluted into four components at 287.0, 285.9, 284.8, and 283.5 eV corresponding to O–C=O, C–C, C=C, and C–Si bonds, respectively. The presence of the C-Si bond further confirms the chemical composition of the SiC shell. Also, the appearance of C=C and C–C bonds reflects the facts that C diffused into the NiSi catalyst alloys during nucleation. The dependence of the integrated intensity of the observed chemical bonds on Ni thickness is displayed in Figure 5f. It can be found that Si–Si content in the nanowires is insignificant and at the Ni thickness of 270 nm it is totally disappeared. By increasing the Ni thickness from 110 to 270 nm, Ni–Si incorporation is decreased while that of Si–O_x_ increased reflecting the fact that high Ni thickness could induce oxide phase rather than NiSi one. This is probably due to slow diffusion of Ni particles to Si and, hence, oxidation of free or unsaturated bonds on the core of the nanowires. Consequently, SiC incorporation as the shell of nanowires is decreased and SiO_2_ is formed instead at Ni thickness of 270 nm. 

**Figure 6:**
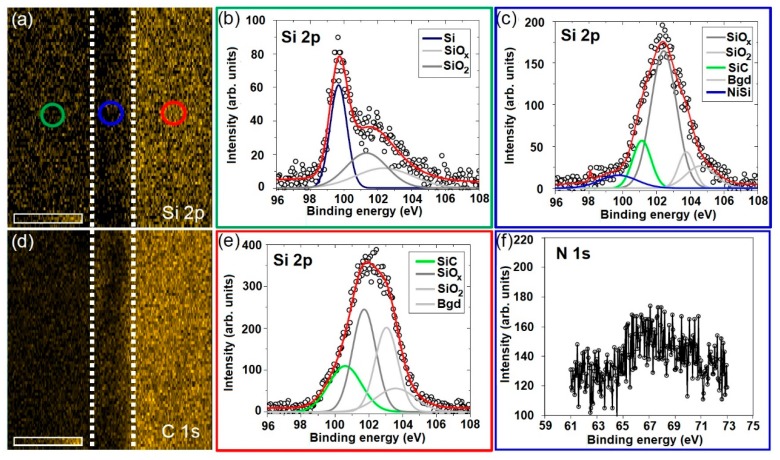
(**a**,**d**) XPS map images of Si 2p and C 1s of NiSi/SiC core-shell nanowires grown by HWCVD, respectively. The Si 2p signal was collected by a higher binding energy detector. The brightness of the map images is relatively corresponding to the intensity of the XPS signals (Si 2p and C 1s). The pixel size is approximately 2 × 2 µm^2^. (**b**,**c**,**e**) The respective XPS narrow-scans of Si 2p at the position in (**a**) indicated by the empty circle with different colors. (**f**) The respective XPS narrow-scan of Ni 3p at the position in (**a**) as indicated by the blue color of the empty circle.

Figure 6a,d shows the SPEM images of Si 2p and C ls core level binding energies, respectively acquired by the higher photoelectron energy channels. These binding energy images were collected on the cross-section of the sample including the c-Si substrate (green, blue and red cycles are representing for the substrate, nickel layer and the nanowires, respectively), the deposited layer and the core-shell nanowires were in good spatial agreement with the SEM image shown in Figure 2e. The SEPM images of Si 2p and C 1s indicate high densities of Si and C on the core-shell nanowires. The XPS narrow-scan spectra of Si 2p and Ni 3s with their deconvoluted components are depicted in Figure 6b–f. The presence of Si and Si oxidation layers on the cross-section of c-Si substrate is verified by the Si 2p peak as shown in Figure 6b. Figure 6c depicts that the deposited layer contained high percentage of SiO*_x_* (64.7%) accompanying with small amounts of NiSi (8.7%) and SiC (16.1%). The large amount of SiO*_x_* in the deposited layers reveals the oxidation of the deposited layers during the nucleation of NiSi particles. The diffusion of Ni into the deposited layer and high affinity of the metallic particles to the residual oxygen could easily result in the oxidation of the deposited layer. The formation of the SiO*_x_* prevents the coalescence of the NiSi particles and it could be essentially important for the growth of NiSi/SiC core-shell nanowires via the NiSi particles [32]. The appearance of Si–C bond indicates the formation of SiC during the nucleation of the NiSi particles. This finding strongly agrees with the radial formation of SiC on the NiSi particles and subsequently mediates as a shell of the NiSi core consistently along the length of the nanowires without any significant taper morphology. Figure 6e shows a typical Si 2p peak of the core-shell nanowires containing of SiC (25.7%) and SiO*_x_* (41.4%). This oxidation states are possibly ascribed to the formation of amorphous SiO*_x_* layer on the nanowires due to the oxidation after the nanowires were exposed to the atmospheric ambient [33]. On the other hand, a little amount of Ni was observed in Ni 3s as shown in Figure 6f owing to the less sensitivity of the photoelectron energy channels to Ni element.

**Figure 7:**
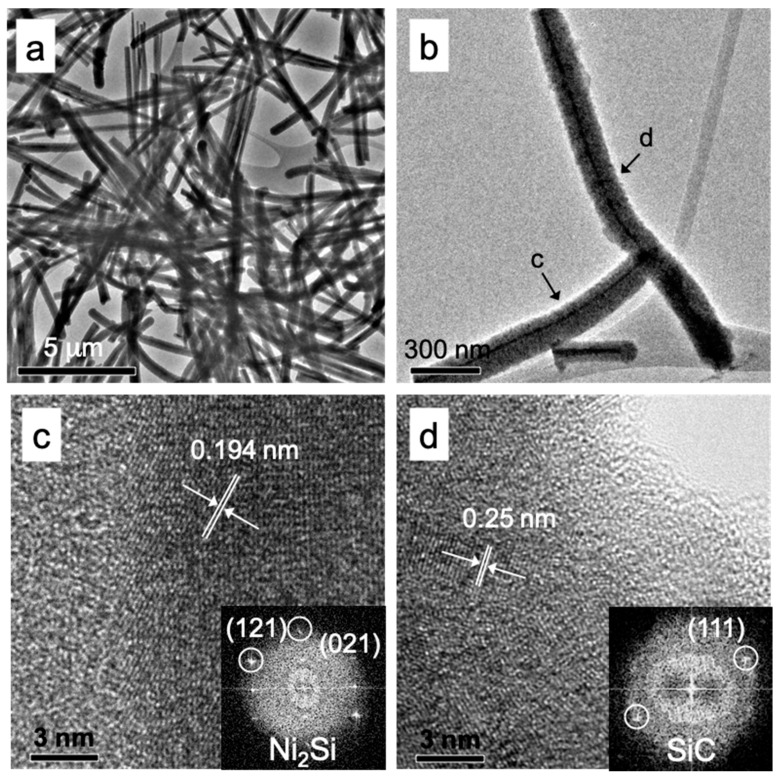
(**a**) Typical TEM image of nanowires grown by HWCVD. (**b**) TEM image of a branched nanowire grown by HWCVD. (**c**,**d**) High-resolution TEM images taken on the nanowire where the positions are indicated by the arrows as shown in (**b**). Insets (**c**,**d**) represent the FFT images of the core and shell of the nanowire respectively.

The microstructure of a typical single NiSi/SiC core-shell nanowire was investigated by TEM and HRTEM images as shown in Figure 7. The typical pile of nanowires in TEM image is illustrated in Figure 7a. The inner structure of the nanowires is clearly revealed in the image. Overall, no noticeable catalyst droplets could be seen on the tip of the nanowires in the image suggesting that the conventional VLS growth mechanism for 1D nanostructures can be simply neglected for these core-shell nanowires [34]. A branched nanowire in TEM image clearly depicts a core-shell structure of a single nanowire as shown in Figure 7b. The core and shell were consistently along the length of the nanowire. The average core diameter and shell thickness were measured about 11 ± 2 and 59 ± 10 nm, respectively. Furthermore, this branched nanowire is apparently grown at the stem of the nanowire resulting from the out-diffusion of the NiSi core and the beginning of the out-diffusion is clearly shown in Figure 7b. Figure 7c depicts a HRTEM image scanned near to the sidewall of the nanowire showing a single crystalline core and an amorphous shell of the nanowire. The estimated lattice spacing is approximately 0.194 nm, corresponding to Ni_2_Si (121) crystallographic plane (JCPDS card no. 065-1507). This suggests that the core nanowires were deposited along the [121] growth direction. The single crystalline of the Ni_2_Si core nanowire was further revealed by a fast Fourier transform (FFT) image as shown in the inset of Figure 7b. The amorphous structure of the shell nanowire is depicted by the HRTEM image collected at the sidewall as shown in Figure 7d. The shell nanowire clearly presents that the amorphous structure consisting of nano-crystallites embedded with its amorphous matrix. These nano-crystallites form as elongated crystallites contributing to the columnar structures as observed in the previous reports [25,35]. The estimated lattice spacing is approximately 0.25 nm corresponding to 3C-SiC (111) crystallographic plane (JCPDS card no. 002-1050). The measured crystallite size is approximately 6.1 ± 0.2 nm. These 3C-SiC nano-crystallites were grown radially toward the nanowire sidewall in [111] growth direction. The crystalline plane of the 3C-SiC (111) was further revealed by a FFT image as shown in the inset of Figure 7d.

**Figure 8:**
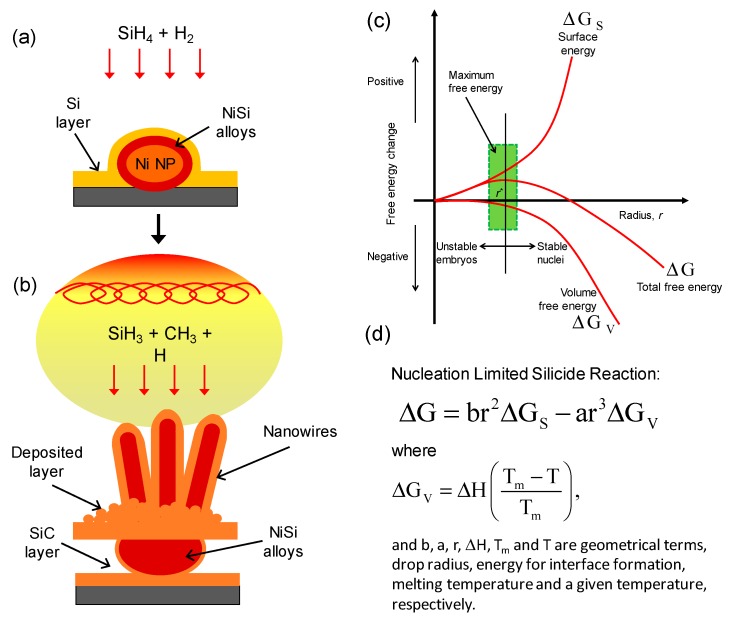
(**a**,**b**) Growth mechanisms of the NiSi nanoparticle and NiSi/SiC core-shell nanowires grown by HWCVD, respectively. (**c**) Free energy changes as function of drop radius for NiSi reactions. The shadowed region is proposed as a region for the growth of NiSi core nanowires. (**d**) The nucleation limited silicide reaction relation for the growth of the core nanowires.

Figure 8 illustrates the schematic diagram of the proposed growth mechanisms of the NiSi nanoparticle and NiSi/SiC core-shell nanowires prepared by HWCVD. In the initial stage of the process, the Ni nanoparticles were formed as a result of the hydrogen plasma treatment at high substrate temperature conditions that similar to the works reported by Alet et al. [36] and Colli et al. [37]. The hydrogen plasma treatment plays an essential role in activating the catalyst metallic nanoparticles [24,38]. In the process, as shown in Figure 8a, SiH_4_ molecules arrive on the Ni nanoparticle surfaces and are catalytically decomposed by the catalyst particles which results in forming of a Si layer on the Ni nanoparticles [39]. Owing to the fast diffusivity of Ni in Si, the formation of NiSi alloys is induced following a solid-state silicide reaction at a substrate temperature above 350 °C [40]. The spontaneous diffusion of Ni into the deposited Si layer could lead to the elongation of NiSi crystals as well as nanowires as reported in the literature [41]. The growth of the nanowires occurred after an increase in the size of the NiSi particles that was larger than a certain critical alloy radius to reduce the surface-free energy of the particles. The subsequent diffusion of the Ni into the NiSi alloys for sustaining the growth of NiSi nanowires eventually turns the Ni nanoparticles completely to large NiSi particles as shown in Figure 3a. In process as shown in Figure 8b, the hot-filament temperature above 1800 °C is capable to decompose the SiH_4_, CH_4_, and H_2_ molecules effectively into the growth precursors of Si and CH_3_. According to Tabata et al. [42], the SiH_4_ molecules were decomposed on the surface of the heating filament following the reaction as SiH_4_ Si + 4H, while the CH_4_ decomposes more effectively by the gas phase reactions of CH_4_ + H CH_3_ + H_2_ [43]. These decompositions generate high densities of the reactive growth precursors of Si arriving on the NiSi particle surfaces thus enhance the growth of NiSi nanowires. Meanwhile, the arrival of CH_3_ on NiSi particle surfaces leads to a surface diffusion of C into the deposited Si layer forming a SiC layer surrounding the NiSi particles and resulting in the core-shell structure. The radial deposition of SiC on the NiSi particles acts as a shell of the particles. The formation of SiC shell layer could form a diffusion barrier that hindering the further diffusion of Ni into the deposited layer [31]. This stimulates the axial growth of nanowires towards vertical. The successive decompositions of SiH_4_ and CH_4_ produce high density of reactive atomic H and, thus, enhance the hydrogen etching effect [44,45]. The reactive hydrogen etching increases the local nucleation sites for the formation of SiC nanograins as observed on the deposited layer shown in Figure 2. The SiC nanograins that formed the deposited layer become thicker with increase in Ni film thicknesses, as well as the size of the NiSi particles as evidenced in Figure 2b,d,f. Apparently, the formation of the SiC crystals is also attributed to the hydrogen etching effect which enhances the nucleation sites and results in the growth of the larger faceted crystals.

The growth of NiSi/SiC core-shell nanowires begins from the nucleation of NiSi solid catalyst particles which is the prerequisite nucleation sites for the initial precipitation of the core nanowires. The nucleation of NiSi was reported to be occurred at a narrow temperature region that strongly depends on the change of NiSi particle sizes in the maximum free energy [46]. Figure 8c illustrates the free energies change as function of drop radius for NiSi reactions in the growth of NiSi core nanowires. The unique growth mechanism for NiSi nucleation is related to the nucleation limited silicide reaction which was described by J. Kim [30]. For the given thermodynamic free energy relations: $\Delta G=br^{2}\Delta G_{S}-ar^{3}\Delta G_{V}$, where $r_{c}=-2\gamma /\Delta G_{V}$, $\Delta G_{V}=-\left(k_{B}T/v\right)\ln\left(P_{saat}/P_{o}\right)$, and ΔG, $\Delta G_{S}$, $\Delta G_{V}$, b, a, r, $r_{c}$, γ, $k_{B}$, v, and $P_{saat}/P_{o}$ are the total free energy, surface free energy, volume free energy, geometrical terms, drop radius, critical drop radius, unit surface free energy, Boltzmann constant, molar volume of the drop, and degree of supersaturation, respectively [47]. According to the free energies change, the change of the NiSi particle size, $r_{c}$, is directly proportional to the degree of supersaturation in the particles at a constant reaction temperature [11]. The reaction of Ni and Si were initially formed NiSi solid particles, and the successive diffusion of Ni into the particles increases the particle size as a result of the tendency to reduce the surface free energy. The particles larger than $r_{c}$ will grow nanowires spontaneously with decreasing in free energy. While for the particle smaller than $r_{c}$, no nanowires will form owing to the insufficient of Ni or the lower temperatures for the Ni and Si reaction. In this case, the supersaturation reaction, $P_{saat}/P_{o}$, is referred to the diffusion rate of Ni into the nucleation sites of the NiSi particles at a fixed reaction temperature. It can be seen that the NiSi particles get larger in size with increase in Ni film thickness as shown in Figure 2b,d,f. These solid catalyst particle sizes are in between 60 to 210 nm which is in the range of the drop radius size for the nucleation limited silicide reactions as reported [29,30,39]. The thicker Ni film increases the diffusion of Ni into the deposited layer resulting to a thicker deposition layer thus decreasing the degree of supersaturation in the particles. Consequently, $r_{c}$ decreases as verified by the $P_{saat}$ at a fixed initial pressure. The expansion of the NiSi particles at the beginning of its nucleation is to reduce the surface free energy for maintaining the total free energy in the maximum free energy region. 

**Figure 9:**
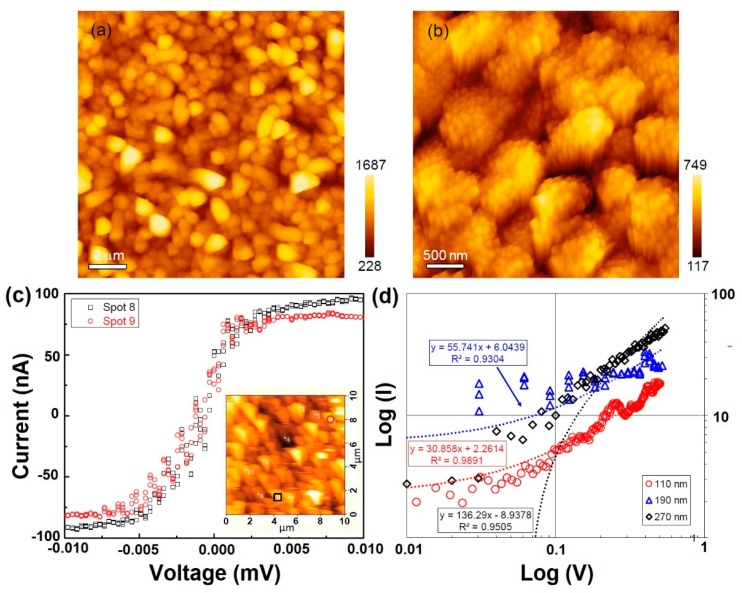
(**a**,**b**) Atomic Force Microscopy (AFM) images of the NiSi/SiC core-shell nanowires grown by HWCVD. (**c**) Current–voltage curves of the nanowires obtained at different spots with the positions as shown in inset of the conducting AFM image. (**d**) Current–voltage curves of the nanowires at different spots in the Ohmic regions.

Figure 9a,b shows the AFM images of the NiSi/SiC core-shell nanowires at different scan sizes. The cluster of the nanowires is clearly observed and their vertical bundle is attributed to their nucleation limited silicide grown reaction. The details of the nanowire growth are obviously shown in Figure 9b. The clustering of nanowires can be observed in the image with the estimated average cluster size of approximately 29 ± 8 nm. The obtained average cluster size is closely consistent with the average diameter of the bundle nanowires measured by the FESEM image as shown in Figure 1c. Figure 9c demonstrates the current–voltage (I–V) curves of the clustered nanowires by a conducting AFM measurement. The selected clusters were identified by the open circle and square box as shown in the inset of Figure 9c. These I–V curves demonstrate a linear behavior at low voltage region. However, the curves turn to rectifying current voltage behavior for the applied voltages above 0.005 mV. The linear I–V characteristic is due to the metallic electrical property of NiSi and the Schottky curve could be attributed to the heterostructure of core-shell nanowire. By fitting the linear region of the I–V curves, the estimated conductivity of the bundle nanowires for the spots 8 and 9 are approximately 1.57 × 10^3^ and 1.44 × 10^3^ Ω^−1^ cm^−1^, respectively, with the scanned area of 0.403 m^2^. Figure 9d demonstrates the I–V curves of the NiSi/SiC core-shell nanowires grown at different Ni film thicknesses of 110, 190, and 270 nm, by the conducting AFM measurements. The linear regions of the I–V curves were fitted in a log scale for obtaining the ohmic contact characteristic of the nanowires and the bottom layer. The estimated conductivities of the single nanowire prepared at Ni film thicknesses of 110, 190, and 270 nm were approximately 4.11 × 10^2^, 1.57 × 10^2^, and 1.36 × 10^2^ Ω^−1^ cm^−1^, respectively which is an order lower compared to that of bundle nanowires owing to the smaller surface area of single nanowire. However, these conductivities of the NiSi/SiC core-shell nanowires are relatively higher than the literature data (10^2^ Ω^−1^ cm^−1^) and suggests that the formation of SiC shell layer on the nanowires could probably improve the electrical performance of the nanowires-based devices for harsh environment applications such as field effect transistors, field emitters, space sensors, and electrochemical devices [10,22]. 

## 4. Conclusions

The well-aligned NiSi/SiC core-shell nanowires by one-step process of HWCVD were presented and their properties were discussed. The nanowires were grown at various Ni thicknesses by heat transfer from a hot filament at 1850 °C. The NiSi solid catalyst particles act as nano-templates for the growth of the nanowires vertically and in well-alignment. The increase in the Ni thickness improve the alignment of the nanowires towards vertically morphologies but reduce the height of the nanowires. The grown nanowires have an average diameter, length, and growth rate of 75.6 nm, 3.1 m, and 10.3 nm/s, respectively and the nanowires were structured by a single-crystalline NiSi and an amorphous SiC as the core and the shell. The shell of the nanowires showed the presence of 3C-SiC nano-crystallites embedded within an amorphous matrix. The precipitation of the NiSi core nanowires was followed by the solid-diffusion control process. Whereas, the formation of SiC shell was attributed to the surface migration of SiC on the NiSi solid catalyst particle surfaces. The roles of SiO_x_ in the deposited layer and NiSi particle size in the growth of these core-shell nanowires were extensively elucidated. The estimated conductivities of the single nanowire prepared at Ni film thickness of 110, 190, and 270 nm were measured as 4.11 × 10^2^, 1.57 × 10^2^, and 1.36 × 10^2^ Ω^−1^ cm^−1^, respectively and relatively higher than the reported literature. The single crystalline NiSi and amorphous SiC heterostructure core-shell nanowires show promising potential applications as a 1D electrode for various electrical and electrochemical applications.

## Figures and Tables

**Table 1:** Elemental contents (%) of the NiSi/SiC core-shell nanowires grown by HWCVD at Ni thickness of 270 nm. The labels of the spots as below are indicated in Figure 4b.

Spot	C	O	Ni	Si
A	25.45	13.72	11.70	48.03
B	17.02	7.46	29.19	45.14

## References

[1] Gupta K., Brahma S., Dutta J., Rao B., Liu C.-P. (2019). Recent progress in microstructure development of inorganic one-dimensional nanostructures for enhancing performance of piezotronics and piezoelectric nanogenerators. Nano Energy.

[2] Yang P. (2011). The Chemistry and Physics of Semiconductor Nanowires. MRS Bull..

[3] Lu Y., Du S., Steinberger-Wilckens R. (2016). One-dimensional nanostructured electrocatalysts for polymer electrolyte membrane fuel cells—A review. Appl. Catal. B Environ..

[4] Chen S.-Y., Yeh P.-H., Wu W.-W., Chen U.-S., Chueh Y.-L., Yang Y.-C., Gwo S., Chen L.-J. (2011). Low Resistivity Metal Silicide Nanowires with Extraordinarily High Aspect Ratio for Future Nanoelectronic Devices. ACS Nano.

[5] Lee C.-Y., Lu M.-P., Liao K.-F., Wu W.-W., Chen L.-J. (2008). Vertically well-aligned epitaxial Ni_31_Si_12_ nanowire arrays with excellent field emission properties. Appl. Phys. Lett..

[6] Roy S., Midya K., Duttagupta S.P., Ramakrishnan D. (2014). Nano-scale NiSi and n-type silicon based Schottky barrier diode as a near infra-red detector for room temperature operation. J. Appl. Phys..

[7] Jiang Y., Li Z., Li B., Zhang J., Niu C. (2016). Ni_3_Si_2_ nanowires grown in situ on Ni foam for high-performance supercapacitors. J. Power Sources.

[8] Chiu W.-L., Chiu C.-H., Chen J.-Y., Huang C.-W., Huang Y.-T., Lu K.-C., Hsin C.-L., Yeh P.-H., Wu W.-W. (2013). Single-crystalline δ-Ni_2_Si nanowires with excellent physical properties. Nanoscale Res. Lett..

[9] Senthilarasu S., Sathyamoorthy R., Lalitha S. (2004). Synthesis and characterization of β-FeSi_2_ grown by thermal annealing of Fe/Si bilayers for photovoltaic applications. Sol. Energy Mater Sol. Cells.

[10] Dasgupta N.P., Xu S., Jung H.J., Iancu A., Fasching R., Sinclair R., Prinz F.B. (2012). Nickel Silicide Nanowire Arrays for Anti-Reflective Electrodes in Photovoltaics. Adv. Funct. Mater..

[11] Lin J.-Y., Hsu H.-M., Lu K.-C. (2015). Growth of single-crystalline nickel silicide nanowires with excellent physical properties. CrystEngComm.

[12] Chen C.Y., Lin C.A., Chen M.J., Lin G.R., He J.H. (2009). ZnO/Al_2_O_3_ core–shell nanorod arrays: Growth, structural characterization, and luminescent properties. Nanotechnology.

[13] Chen C., Shehata S., Fradin C., LaPierre R., Couteau C., Weihs G. (2007). Self-Directed Growth of AlGaAs Core—Shell Nanowires for Visible Light Applications. Nano Lett..

[14] Reddy A.L.M., Gowda S.R., Shaijumon M.M., Ajayan P.M. (2012). Hybrid Nanostructures for Energy Storage Applications. Adv. Mater..

[15] Zekentes K., Rogdakis K. (2011). SiC nanowires: Material and devices. J. Phys. D Appl. Phys..

[16] Shao R., Zheng K., Zhang Y., Li Y., Zhang Z., Han X. (2012). Piezoresistance behaviors of ultra-strained SiC nanowires. Appl. Phys. Lett..

[17] Fan J., Wu X., Chu P. (2006). Low-dimensional SiC nanostructures: Fabrication, luminescence, and electrical properties. Prog. Mater. Sci..

[18] Nazarudin N.F.F.B., Azizan S.N.A.B., Rahman S.A., Goh B.T. (2014). Growth and structural property studies on NiSi/SiC core-shell nanowires by hot-wire chemical vapor deposition. Thin Solid Films.

[19] Chong S.K., Goh B.T., Aspanut Z., Muhamad R.M., Dee C.F., Rahman S.A. (2011). Effect of substrate temperature on gold-catalyzed silicon nanostructures growth by hot-wire chemical vapor deposition (HWCVD). Appl. Surf. Sci..

[20] Binti Hamzan N., bin Ramly M.M., Huang N.M., Rahman S.A., Goh B.T. (2017). Growth of high density NiSi/SiC core-shell nanowires by hot-wire chemical vapour deposition for electrochemical applications. Mater. Charact..

[21] Wagner R., Ellis W. (1964). Vapor-liquid-solid mechanism of single crystal growth. Appl. Phys. Lett..

[22] Kim C.J., Kang K., Woo Y.S., Ryu K.G., Moon H., Kim J.M., Zang D.S., Jo M.H. (2007). Spontaneous Chemical Vapor Growth of NiSi Nanowires and Their Metallic Properties. Adv. Mater..

[23] Nash P., Nash A. (1987). The Ni−Si (Nickel-Silicon) system. Bull. Alloy Phase Diagr..

[24] Goh B.T., Rahman S.A. (2014). Study of the growth, and effects of filament to substrate distance on the structural and optical properties of Si/SiC core—Shell nanowires synthesized by hot-wire chemical vapor deposition. Mater. Chem. Phys..

[25] Qian G., Rahman S., Goh B. (2015). Controlled growth of Si-based heterostructure nanowires and their structural and electrical properties. Nanoscale Res. Lett..

[26] Ramly M.M., Hamzan N., Nazarudin N.F.F., Qian G., Aspanut Z., Rahman S.A., Goh B.T. (2018). Growth of Si-based core–shell nanowires through gases decomposition reactions with tunable morphologies, compositions, and electrochemical properties. J. Mater. Sci.-Mater. EL..

[27] Lu K.-C., Wu W.-W., Wu H.-W., Tanner C.M., Chang J.P., Chen L.J., Tu K.N. (2007). In situ Control of Atomic-Scale Si Layer with Huge Strain in the Nanoheterostructure NiSi/Si/NiSi through Point Contact Reaction. Nano Lett..

[28] Hannon J.B., Kodambaka S., Ross F.M., Tromp R.M. (2006). The influence of the surface migration of gold on the growth of silicon nanowires. Nature.

[29] Kim J., Anderson W.A. (2005). Spontaneous nickel monosilicide nanowire formation by metal induced growth. Thin Solid Films.

[30] Kim J. (2012). Thermodynamic mechanism of nickel silicide nanowire growth. Appl. Phys. Lett..

[31] Tong Goh B., Abdul Rahman S. (2014). Synthesis of nickel catalyzed Si/SiC core–shell nanowires by HWCVD. J. Cryst. Growth.

[32] Kang K., Kim S.-K., Kim C.-J., Jo M.-H. (2008). The Role of NiOx Overlayers on Spontaneous Growth of NiSix Nanowires from Ni Seed Layers. Nano Lett..

[33] Al-Masoodi A.H.H., Hamzan N.B., Al-Masoodi A.H.H., Rahman S.A., Goh B.T. (2016). Influences of hydrogen dilution on the growth of Si-based core–shell nanowires by HWCVD, and their structure and optical properties. Appl. Phys. A.

[34] Panciera F., Chou Y.C., Reuter M.C., Zakharov D., Stach E.A., Hofmann S., Ross F.M. (2015). Synthesis of nanostructures in nanowires using sequential catalyst reactions. Nat. Mater..

[35] Hamzan N.B., Nordin F.N.B., Rahman S.A., Huang N.M., Goh B.T. (2015). Effects of substrate temperature on the growth, structural and optical properties of NiSi/SiC core-shell nanowires. Appl. Surf. Sci..

[36] Alet P.-J., Eude L., Palacin S., Cabarrocas P.R.I. (2008). Transition from thin gold layers to nano-islands on TCO for catalyzing the growth of one-dimensional nanostructures. Phys. Status Solidi A.

[37] Colli A., Fasoli A., Beecher P., Servati P., Pisana S., Fu Y., Flewitt A., Milne W., Robertson J., Ducati C. (2007). Thermal and chemical vapor deposition of Si nanowires: Shape control, dispersion, and electrical properties. J. Appl. Phys..

[38] Chong S.K., Goh B.T., Dee C.F., Rahman S.A. (2013). Effect of substrate to filament distance on formation and photoluminescence properties of indium catalyzed silicon nanowires using hot-wire chemical vapor deposition. Thin Solid Films.

[39] Lavoie C., d’Heurle F.M., Detavernier C., Cabral C. (2003). Towards implementation of a nickel silicide process for CMOS technologies. Microelectron. Eng..

[40] Hsu H.-F., Huang W.-R., Chen T.-H., Wu H.-Y., Chen C.-A. (2013). Fabrication of Ni-silicide/Si heterostructured nanowire arrays by glancing angle deposition and solid state reaction. Nanoscale Res. Lett..

[41] Zhihong L., Hui Z., Lei W., Deren Y. (2008). Controlling the growth and field emission properties of silicide nanowire arrays by direct silicification of Ni foil. Nanotechnology.

[42] Tabata A., Komura Y. (2007). Preparation of nanocrystalline cubic silicon carbide thin films by hot-wire CVD at various filament-to-substrate distances. Surf. Coat. Technol..

[43] Dasgupta A., Huang Y., Houben L., Klein S., Finger F., Carius R. (2008). Effect of filament and substrate temperatures on the structural and electrical properties of SiC thin films grown by the HWCVD technique. Thin Solid Films.

[44] Kherodia A., Kheraj V., Panchal A.K. (2018). Effects of Hydrogen-Dilution on Opto-Structural Properties of Hot-wire CVD Grown a-Si:H/nc-Si:H Multilayer for Photovoltaics. Silicon.

[45] Yusuke K., Akimori T., Tomoki N., Masaki K., Akihiro K., Teruyoshi M. (2007). Film Properties of Nanocrystalline 3C–SiC Thin Films Deposited on Glass Substrates by Hot-Wire Chemical Vapor Deposition Using CH 4 as a Carbon Source. Jpn. J. Appl. Phys..

[46] Chou Y.-C., Tang W., Chiou C.-J., Chen K., Minor A.M., Tu K.N. (2015). Effect of Elastic Strain Fluctuation on Atomic Layer Growth of Epitaxial Silicide in Si Nanowires by Point Contact Reactions. Nano Lett..

[47] Gambino J., Colgan E. (1998). Silicides and ohmic contacts. Mater. Chem. Phys..

